# Spermidine potentiates anti-tumor immune responses and immunotherapy sensitivity in breast cancer

**DOI:** 10.7150/jca.113235

**Published:** 2025-07-28

**Authors:** Xinyu Yang, Yaxin Feng, Ruxin Wang, Xin Zeng, Boying Gao, Penghan Huang, Huiping Chen, Wenfeng Zeng

**Affiliations:** 1 Guangdong Provincial Key Laboratory of Malignant Tumor Epigenetics and Gene Regulation, Guangdong-Hong Kong Joint Laboratory for RNA Medicine, Breast Tumor Center, Sun Yat-Sen Memorial Hospital, Sun Yat-Sen University, Guangzhou 510120, China.; 2 Breast Tumor Center, Sun Yat-Sen Memorial Hospital, Sun Yat-Sen University, Guangzhou, 510120, China.

**Keywords:** tumor metabolism, spermidine, anti-tumor immunity, CD8^+^ T cell

## Abstract

Spermidine metabolism influences tumor progression and anti-tumor immunity, thereby affecting treatment sensitivity. However, the precise role and therapeutic potential of spermidine in breast cancer remain unclear. Integrated multi-omics analyses (bulk and single-cell RNA sequencing) revealed a significant positive correlation between intratumoral spermidine abundance and immunophenotypic markers of CD8^+^ T cell infiltration and activation (GZMB^+^CD8^+^ T cells). Immunohistochemical and multiplexed immunohistochemistry validation (IHC/mIHC) demonstrated that breast cancer specimens with elevated spermidine production exhibited increased numbers of activated CD8⁺ T cells. Exogenous supplementation with spermidine promoted CD8⁺ T cell activation directly. Furthermore, supplementing spermidine *in vivo* promoted anti-tumor immune responses and enhanced sensitivity to anti-PD-1 immunotherapy combined with chemotherapy. Our findings indicate that boosting spermidine metabolism is a promising strategy to reinvigorate CD8⁺ T cell function and improve the efficacy of checkpoint blockade immunotherapy.

## Introduction

Over the past few decades, researchers have recognized that malignant tumor involves not only intricate genetic and hereditary mechanisms but also the complex interplay of various cells, impacting multiple organ systems 1, 2. Immunocytes, as an important component of the tumor microenvironment (TME), play a significant role in tumorigenesis and development. The effect of anti-tumor immunity in the treatment of malignant tumors has received increasing attention 3. Immune cells participate in the modulation of tumor biological behaviors through various factors such as cytokine secretion and direct intercellular contact 4, 5. The immune cells infiltrated in tumors exhibit both anti-cancer and cancer-promoting effects. These immune cells have the ability to eliminate tumor cells through direct contact or the release of perforin, granzymes, and other factors 6, 7. Conversely, they can also promote tumor progression by producing cytokines that stimulate tumor proliferation and migration 8. However, given the complexity and heterogeneity of tumor, some cunning cancer cells could evade the immune system 9. Understanding the mechanism of tumor immune escape and seeking intervention strategies is crucial to development of tumor treatment strategies 10-12.

Mounting evidence suggests that metabolic reprogramming in tumor-immunity interactions is a crucial factor influencing tumor progression, including anti-tumor immune responses 12, therapeutic responses 13, tumorigenesis and survival 14, 15. Polyamine, as an important part of tumor metabolism 16, 17, are involved in the regulation of cell proliferation, differentiation, and senescence, primarily consisting of spermine, spermidine, and putrescine. Interestingly, investigations demonstrate that polyamine metabolism may present as a therapeutic target in malignancy treatment. 18, 19.

In the complex polyamine family, spermine and spermidine, as classical metabolic checkpoints of cell homeostasis 17, are potentially involved in tumor immune escape and immunotherapy sensitivity 19. However, the precise mechanisms by which spermidine metabolism influences anti-tumor immunity and its therapeutic potential for immunotherapy in breast cancer remain largely unknown.

To bridge this gap in understanding, we have initiated a comprehensive investigation into the effects of spermidine on anti-tumor immunity in breast cancer. Our research methodology encompasses a multifaceted strategy, including transcriptome analysis, single-cell sequencing to explore the impact of spermidine on immune cells, fluorescent imaging of breast cancer tissues, and *in vivo* immunotherapy studies in tumor-bearing mice supplemented with spermidine to validate the effects of spermidine on immune cells. By leveraging these advanced techniques, we aim to uncover the complex interplay between spermidine metabolism and anti-tumor immune responses in breast cancer, thus enabling the development of novel therapeutic strategies that leverage immunomodulatory properties of spermidine to improve cancer treatment outcomes.

## Materials and Methods

### RNA-seq data collection and processing

The transcriptome data of breast cancer patients were obtained from publicly available databases. RNA-seq [RNA-seq; fragments per kilobase million (FPKM)] datasets for breast cancer patients were sourced from the GEO database (https://www.ncbi.nlm.nih.gov/geo/). The transcript per million values from TCGA were converted to FPKM values. Clinical information for the patients, including age, T stage, N stage, and other relevant details, was also collected. Samples were segmented into high/medium/low polyamine activity tertiles according to the FPKM ratio between ornithine decarboxylase 1 (ODC1, synthesis rate-limiting enzyme) and spermidine/spermine N1-acetyltransferase (SSAT, catabolic enzyme).

### Immune cell infiltration analysis

CIBERSORT is an analytical tool capable for estimating 22 types of immune cell infiltration (ICI) within tumors through 500 permutations^20^. The CIBERSORT algorithm, available within the R package, was employed to quantify the level of ICI in breast cancer tissue. Patients are classified based on their polyamine expression levels as described above.

### scRNA-seq data processing

Downstream analysis of scRNA-seq data, sourced from GEO, GSE176078^21^, was conducted using the Seurat (v 4.3.0) and Harmony (v 1.2.0) packages in R (v 4.2.1). The AddModuleScore function from the R package Seurat was utilized to incorporate and add genes from the GOBP_SPERMIDINE_METABOLIC_PROCESS dataset (https://www.gsea-msigdb.org/gsea/msigdb/human/geneset/GOBP_SPERMIDINE_METABOLIC_PROCESS.html) into the GSE176078 single-cell sequencing data. Based on the weighted average of this data, patients were categorized into two groups: high spermidine metabolism in cancer cells (high: CID45171, CID4530N, CID4535, CID4523, CID4066, CID3921, CID3948, CID44991, CID3941, CID4513) and low spermidine metabolism (low: CID4471, CID4290A, CID3963, CID4067, CID4465, CID44971, CID4515, CID4461, CID4495, CID4463). The Seurat R package was implemented for single-cell data preprocessing, while cell annotations for both datasets were annotated according to the original paper or annotation references provided in online data.

### Differential gene analysis

Differentially expressed genes (DEGs) analysis in lymphocyte subsets was conducted using Seurat's FindMarkers function, contrasting samples with high and low spermidine metabolism in cancer cells. The ggplot2 R package (H. Wickham. ggplot2: Elegant Graphics for Data Analysis. Springer-Verlag New York, 2016.) was employed to visualize the DEGs and generate volcano plots. Additionally, the ggrepel package was used to display the gene names of the DEGs with p-values < 0.05 and log2 fold changes (log2FC) > 0.5.

### GO enrichment analysis

Biological process (BP) enrichment analysis of differentially expressed genes was performed using the ClusterProfiler R package 1. The significance level α was set at 0.05, and the Benjamini & Hochberg method was selected for p-value correction, which involves adjusting each p-value and converting it to a q-value. The formula for q-value calculation is q = p * n / rank, where 'rank' refers to the order of p-values after sorting them from smallest to largest. This process yielded the Gene Ontology (GO) analysis results.

### Immunohistochemistry (IHC)

After deparaffinization and antigen retrieval with EDTA at pH 9.0, Sections underwent three 5-minute PBS washing cycles. To eliminate endogenous peroxidase activity and reduce non-specific background signals, the sections were treated with 3% hydrogen peroxide solution for 20 minutes. Following this, the sections were washed again with PBS three times, each for 5 minutes, and then blocked with PBS containing 5% BSA at room temperature for 30 minutes to further reduce background signals. After blocking, the primary antibody against spermidine (abcam, ab7318) was added and incubated overnight at 4°C on a shaker. The sections were then rinsed three times in PBS (5 min each). Next procedures were performed using an immunohistochemistry kit (ZSGB-BIO, ZLI-9017) according to the manufacturer's instructions. The sections were then counterstained with hematoxylin, dehydrated, and mounted in neutral resin. Imaging was performed using a digital pathology slide scanner (KF-FL-120) in brightfield mode. The H-score (histochemical score) was calculated to semi-quantitatively assess protein expression levels in immunohistochemical (IHC) staining. For each tissue section, five representative high-power fields (HPFs, 400× magnification) were randomly selected. The staining intensity was quantified according to the following grading scale: 0 (no staining), 1+ (weak), 2+ (moderate), and 3+ (strong). The percentage of positively stained cells within each intensity category was independently evaluated by two blinded pathologists. The H-score was computed using the formula:

H-score=(1+ cells×1)+(2+ cells×2)+(3+cells×3)

where the percentage values for each intensity category (ranging 0-100%) were multiplied by their respective weighting factors. Final scores ranged from 0 to 300, with higher values indicating stronger protein expression. Discordant evaluations (<15% score variance) were resolved by consensus review, while cases with larger discrepancies were reanalyzed using a multi-head microscope.

### Multiplex immunohistochemical (mIHC)

The deparaffinization and antigen retrieval steps for sections are consistent with those used in immunohistochemistry. For immunofluorescence, there is no need to eliminate endogenous peroxidase before proceeding directly to the blocking step. The sections were incubated overnight at 4°C with anti-spermidine antibody (abcam, ab7318), followed by three 5-minute PBS washes. The multiple immunofluorescence staining kit (absin, abs50014) was used to visualize the fluorescent signals according to the manufacturer's instructions. Following another round of antigen retrieval and blocking steps, subsequent antibody incubations were performed for granzyme B (abcam, ab255598) and CD8 (abcam, ab237709), with steps similar to those previously described. The nuclei were counterstained with DAPI (Servicebio, G1012) at room temperature for 15 minutes in the dark. Imaging was subsequently performed using the KFBIO digital pathology slide scanner KF-FL-400. For TUNEL staining, tissue sections were first incubated overnight at 4°C with a pan CK monoclonal antibody (Abcam, ab7753; 1:200 dilution in blocking buffer). After PBS washes (3 × 5 min), apoptosis was detected using a TUNEL assay kit (Beyotime, C1086) following the one-step protocol. Secondary staining employed a donkey anti-mouse IgG-Alexa Fluor 555 (1:500; Invitrogen, A31572) for 1 h at room temperature. Finally, DAPI nuclear counterstaining and mounting were conducted as described in the previous protocol.

### Cell staining and flow cytometry

Cells were collected into tubes, and a washing step was performed with phosphate-buffered saline (PBS) solution. First, cells were incubated with FcR Blocking Reagent (Miltenyi Biotec, 130-059-901) to block nonspecific binding. Cell viability was assessed by 15-minute staining with FVD780 (eBioscience, 65-0865-14) at room temperature under dark conditions. Then, antibodies against membrane molecules, including Alexa Fluor 700 anti-human CD3 antibody (Biolegend, 300323), FITC anti human CD8 antibody (Biolegend, 300906) were used to stain cells for 15min at room temperature without light exposure. For intracellular molecules, cells were fixed using fixation buffer (Biolegend, 420801) for 15min at room temperature, followed by two washes with perm/wash buffer (BioLegend, 421002). Subsequently, cells were incubated with intracellular antibodies in wash buffer for 3 hours protected from light, including: PE/Cyanine7-conjugated anti-human IFN-γ (BioLegend, 502527), Brilliant Violet 421™ anti-human/mouse Granzyme B (BioLegend, 515409). For ki67 staining, cells were fixed and permeabilized using True-Nuclear™ Transcription Factor Buffer Set (Biolegend, 424401). The cells were mixed with intracellular antibodies in perm buffer including PE anti-mouse/human Ki-67 Antibody for 3 hours, and flow cytometric analysis was conducted after two washes with PBS. Cells were acquired on CytoFLEX S (Beckman, USA). Further analysis of the data was performed with FlowJo V.10.6.2 software.

### *In vivo* spermidine supplementing experiment

To establish a subcutaneous tumor-bearing mouse model, we first amplified EO771 cells (CH3 Biosystems, 940001) overexpressed with luciferase (EO771-luc) *in vitro* for no more than five passages. Female C57BL/6J wild-type mice, aged 6 weeks, were selected for the experiment, and 1×10^6 EO771-luc cells resuspended in 100 ml of PBS were implanted into the fourth pair of mammary fat pads using a syringe. Chemotherapy and immunotherapy treatments were initiated one week after tumor inoculation in mice. Docetaxel (Sanofi Mature IP) was administered intraperitoneally at 10 mg/kg one week after tumor inoculation in mice, followed by a single intraperitoneal injection of PD-1 antibody (SYD, PA007162) at a dosage of 10 mg/kg. Mice supplemented with spermidine received peritumoral injection of spermidine (sigma, S0266) at a dosage of 5 mg/kg every 3 days after chemotherapy and immunotherapy. The mice of oral administration group received the drug via oral gavage at a dose of 10 mg/kg per mouse, with the same dosing schedule as the peritumoral injection group. During the experiment, tumor growth was observed every three days, and tumor size was measured using a vernier caliper. The animal experiment was terminated after three weeks of treatment or when the maximum tumor diameter reached 1.5 cm. Tumor volume was calculated using the formula Volume (mm³) = (length × width × width) / 2, and a growth curve was plotted. For additional investigations, tumor specimens were fixed in paraformaldehyde, embedded in paraffin, and sliced into sections for immunofluorescence staining. The Institutional Animal Care and Use Committee at Sun Yat-Sen University granted approval for animal experiments (ethical number: 2023002272).

### Statistical analysis

Statistical analyses were performed using GraphPad Prism 9.0. Data normality was first assessed with the Shapiro-Wilk test. For comparisons between two groups with normally distributed datasets, Student's t-test was applied to evaluate statistical differences. Statistical comparisons among multiple groups were performed using one-way ANOVA followed by Holm-Šidák multiple comparisons correction. Parametrically analyzed data are presented as mean ± standard deviation (s.d.), while non-normally distributed data were analyzed using the Mann-Whitney U test (non-parametric rank-sum test) and expressed as median with 95% confidence interval (CI). p value less than 0.05 was considered statistically significant. The significance levels were indicated as follows: ns, no significance, *p < 0.05, **p < 0.01, ***p < 0.001, ****p < 0.0001.

## Results

### Spermidine enrichment was correlated with enhanced anti-tumor immunity in breast cancer patients

The synthesis of polyamines involves several key steps (Figure S1): (1) Initial decarboxylation: The first step involves the decarboxylation of L-ornithine (Orn), an intermediate derived from L-arginine (Arg) via arginase 1 (ARG1). (2) Putrescine formation: Orn is converted to putrescine by the rate-limiting enzyme ornithine decarboxylase 1 (ODC1). (3) Spermidine synthesis: Putrescine is subsequently converted to spermidine by spermidine synthase (SPDS). (4) Spermine production: Spermidine is further converted to spermine by spermine synthase (SPMS). (5) Catabolic regulation: Polyamine degradation involves the retroconversion of higher polyamines (spermine → spermidine → putrescine), primarily mediated by spermidine/spermine N¹-acetyltransferase (SSAT)-catalyzed acetylation 22.

**Figure 1:**
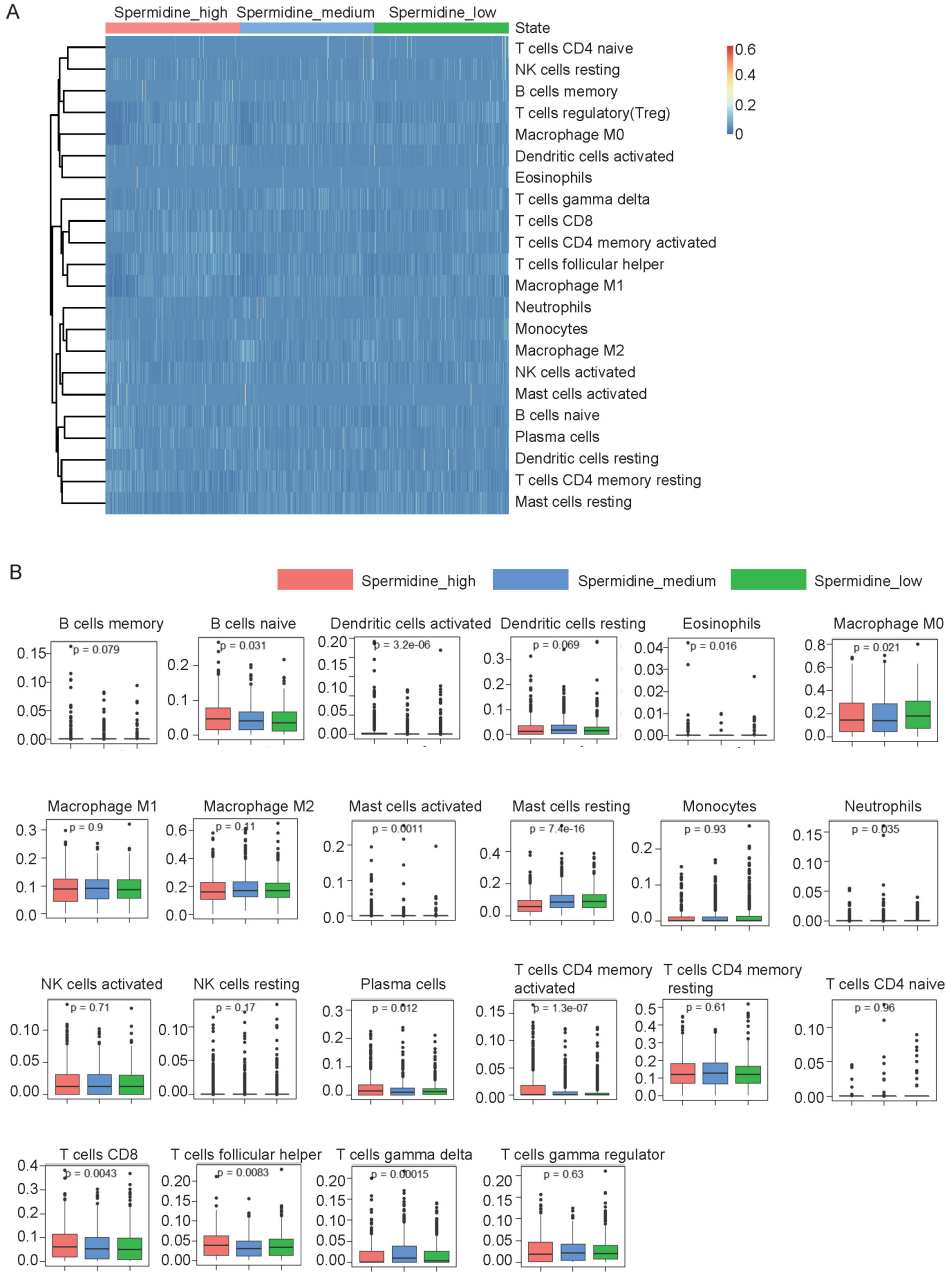
** Spermidine enrichment was correlated with enhanced anti-tumor immunity in breast cancer patients. (A)** The results of immune cell infiltration (ICI) analysis using CIBERSORT on high-spermidine-enrichment, middle-spermidine-enrichment and low-spermidine-enrichment transcriptome data from breast cancer patients in TCGA dataset. **(B)** Box plot displaying the differences in ICI among the high-spermidine-enrichment, middle-spermidine-enrichment and low-spermidine-enrichment breast cancer samples.

The immune activation status of breast cancer patients was determined by the analysis of transcriptome sequencing data of the primary breast cancer samples from the TCGA database. We calculated the spermidine enrichment score for each patient as the ratio of ornithine decarboxylase (ODC1) expression level, which plays a crucial role in spermidine synthesis, to the expression level of spermidine degrading enzyme (SSAT). Based on the spermidine enrichment score, we divided the TCGA breast cancer primary focus data into three equal groups: high spermidine enrichment, middle spermidine enrichment and low spermidine enrichment. The level of anti-tumor immunity in each TCGA breast cancer sample was determined using the CIBERSORT method and the deconvolution algorithm that had been trained to predict the composition and function of immune cells in the tumor through the deconvolution of transcriptome sequencing data. We show the anti-tumor immunity level of three groups of samples with high, middle and low spermidine enrichment levels (Figures 1A and 1B). The results suggested that breast cancer patients with high levels of spermidine enrichment possess more and stronger anti-tumor effector lymphocytes (Figures 1A and 1B). More importantly, we observed that CD8^+^ T cells, which play a crucial role in anti-tumor immunity, were more abundant in the high spermidine enrichment group compared to the other two groups (Figure 1B). This finding suggested that spermidine might affect anti-tumor immunity by influencing CD8^+^ T cell infiltration.

### Spermidine hypermetabolism was associated with the activation of CD8^+^T cells

**Figure 2:**
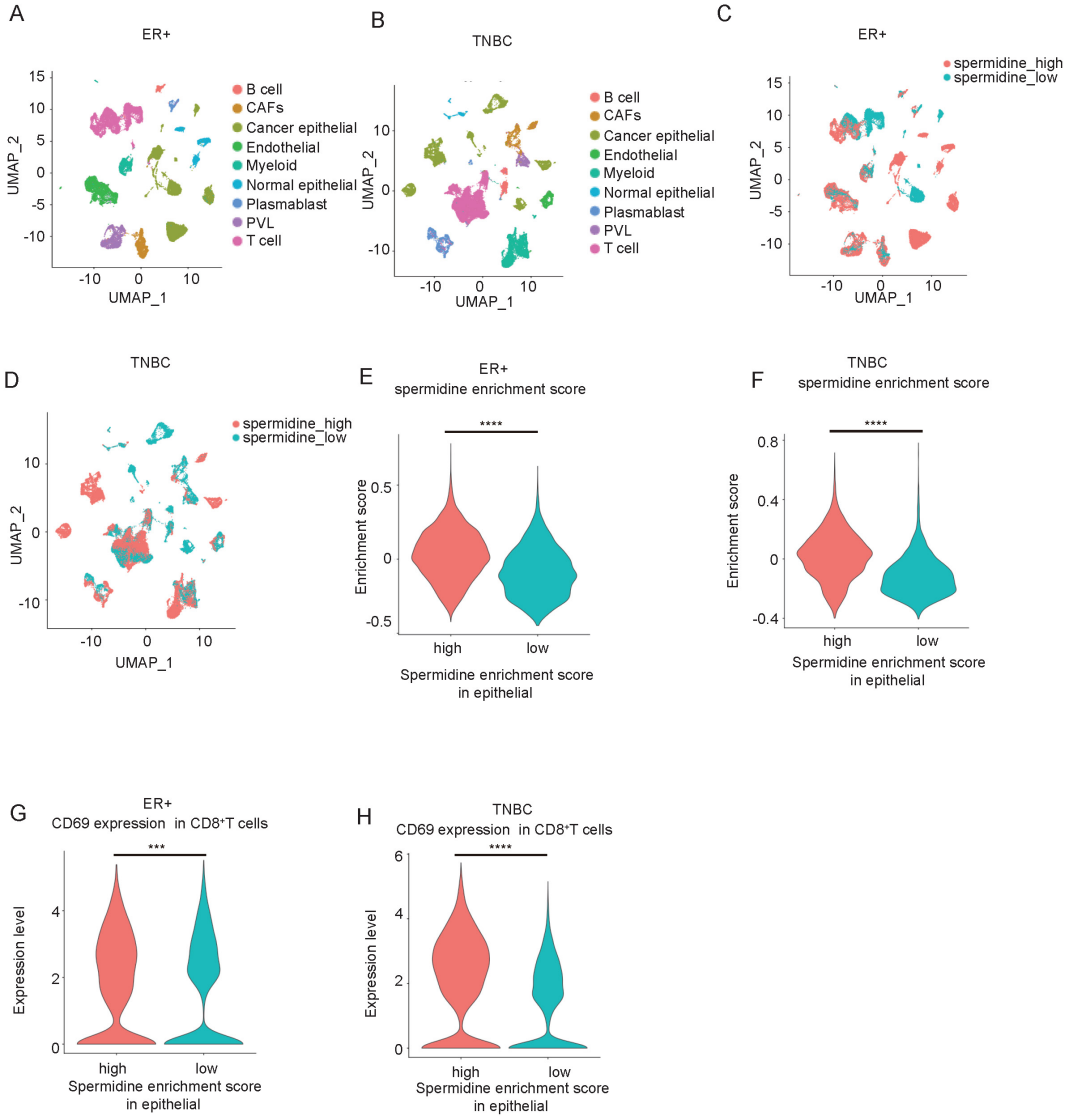
** Spermidine hypermetabolism was associated with the activation of CD8^+^ T cells. (A-B)** UMAP visualization of ER^+^ breast cancer samples (A, *n* = 11) and TNBC samples (B, *n* =10), analyzed by scRNA-seq from GSE176078. Clusters were annotated for their cell types according to the original literature. **(C-D)** UMAP visualization of all epithelial cells from ER^+^ breast cancer samples **(C)** and TNBC samples **(D)**, colored by high or low spermidine enrichment score. **(E-F)** Violin plot visualization of high and low spermidine enrichment scores in breast cancer samples. Statistical significance is denoted by “****”, indicating p < 0.0001 as determined by unpaired two-tailed Student's t-test. **(G-H)** Violin plot visualization of the CD69 expression levels in CD8^+^ T cells between the breast cancer samples with high or low spermidine enrichment score. Statistical significance is denoted by “****”, indicating p < 0.0001 as determined by unpaired two-tailed Student's t-test.

As previously mentioned, spermidine metabolism may impact the tumor immune infiltration status. To further analyze the effects of spermidine metabolism on different immune cells, we analyzed scRNA-seq data from GEO (GSE176078), ensuring the quality of sequenced cell samples based on gene density and unique molecular identifier (UMI) captured at the single-cell level. The dataset included single-cell sequencing results from 26 cases of primary breast cancer tissues without chemotherapy, comprising 11 ER-positive, 5 HER2-positive, and 10 triple-negative breast cancer (TNBC) cases, along with cellular annotation information. We excluded 6 samples without annotated cancer cell populations, and performed group analysis in ER^+^ and TNBC subgroups with a larger number of cases. Dimensionality reduction and cluster annotation were conducted according to the annotations in the original literature 21 (Figures 2A and 2B). Based on the comprehensive scores of cancer cells in each sample from the spermidine metabolism dataset, we divided the samples into spermidine hypermetabolism and spermidine hypometabolism groups using the median score as the cut-off value (Figures 2C and 2D). A violin plot showed the significant differences in spermidine metabolism levels between these two groups in ER^+^ and TNBC samples (Figures 2E and 2F). Concurrently, breast cancer samples with high spermidine metabolism showed a remarkable increase in the expressing levels of T-cell activation and effector-related gene CD69 in CD8^+^ T cells (Figures 2G and 2H). These findings suggested that the level of spermidine metabolism in breast cancer cells exerted significant influence on the gene expression and function of CD8^+^ T cells.

### Breast cancer cells produced abundant spermidine

**Figure 3:**
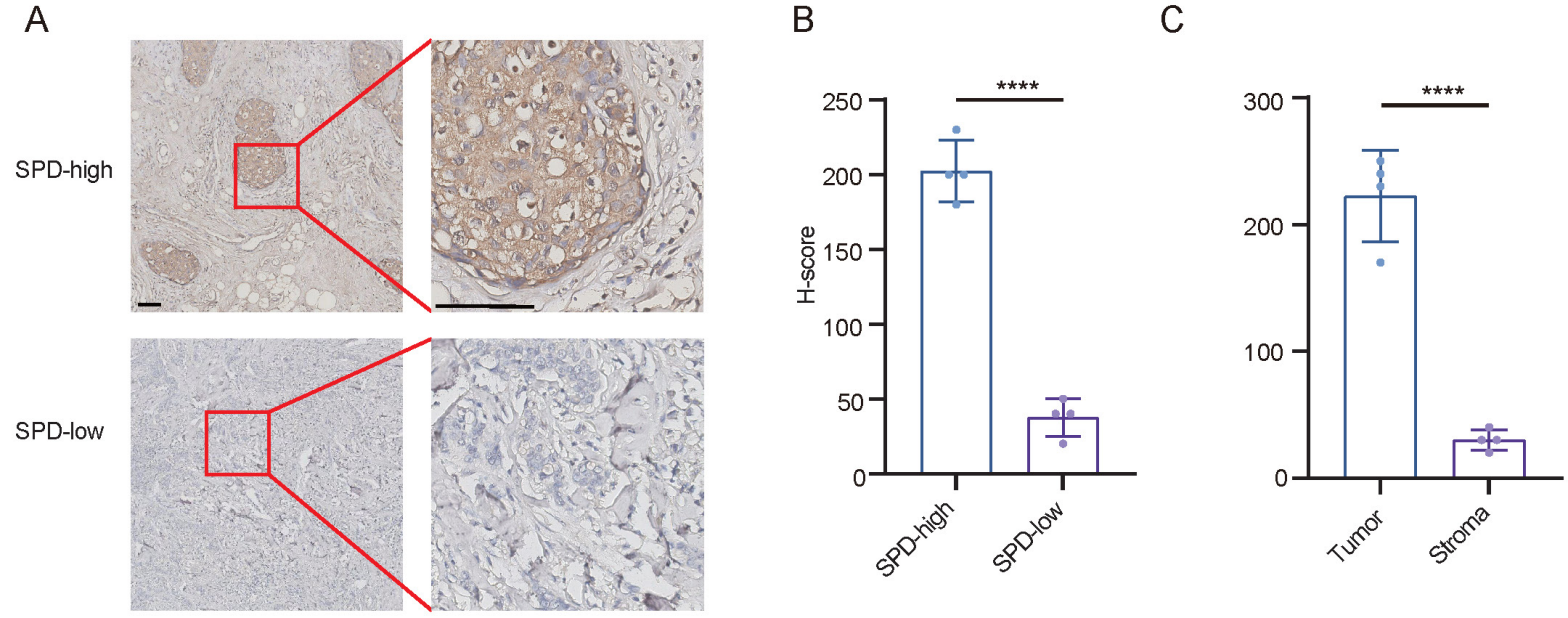
** Breast cancer cells produced abundant spermidine. (A)**Representative images of IHC staining for spermidine (SPD) in breast cancer samples. Scale bars: 100 mm. **(B)**H-scores of spermidine IHC staining, n = 4, Data are mean ± standard deviation (SD). Statistical significance is denoted by “****”, indicating p < 0.0001 as determined by unpaired two-tailed Student's t-test. **(C)**H-scores of spermidine IHC staining within neoplastic cellular areas versus stromal compartments, n=4, Values represent mean ± standard deviation (SD). The symbol "****" denotes statistical significance with p < 0.0001, evaluated using unpaired two-tailed Student's t-test.

In addition to the analysis of single cell transcriptome, we found that breast cancer cells are contributors to spermidine metabolism. We validated the conclusions drawn from our bioinformatics analysis by analyzing spermidine levels in 8 cases of breast cancer. We performed quantitative analysis of spermidine content across stromal and neoplastic areas in samples classified as high spermidine expression. Our findings demonstrated that malignant cell territories displayed markedly elevated spermidine content relative to the surrounding stromal compartments (Figures 3A-3C).

### The intratumoral accumulation of spermidine was positively correlated with the abundance of activated CD8⁺ T cells

**Figure 4:**
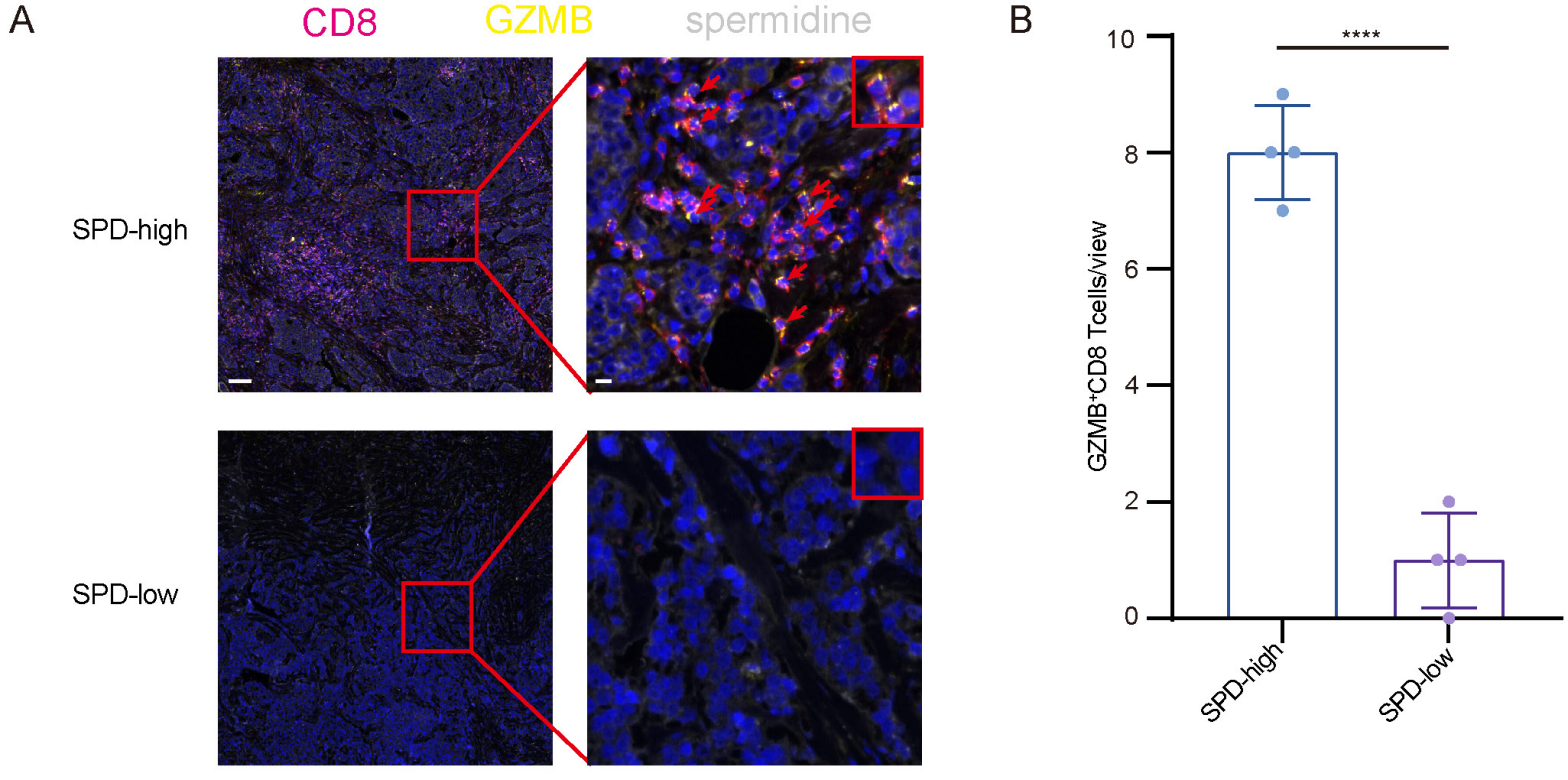
** GZMB^+^ CD8^+^ T cell infiltration had a closer relationship with cancer cells with high levels of spermidine. (A)**Representative images of mIHC staining for CD8 (red), GZMB (yellow), and spermidine (white), in samples of breast cancer tissues with high or low spermidine level. Scale bar: 100 μm (left), 10 μm (right). **(B)**Analysis of the proportion of GZMB+ CD8+ T cells, n=4, Data are presented as mean ± standard deviation (SD). Statistical significance is denoted by “****”, indicating p < 0.0001 as determined by unpaired two-tailed Student's t-test.

Furthermore, through immunohistochemical staining, breast cancer specimens were stratified into cohorts based on spermidine abundance: high and low spermidine enrichment, and observed the activation level and distribution pattern of CD8^+^ T cells. Within specimens exhibiting enhanced spermidine content, we found that CD8^+^ T lymphocytes expressed higher levels of granzyme B, and a higher proportion of CD8^+^ T lymphocytes were granzyme B-positive (Figure 4A and 4B). Meanwhile, the activated CD8^+^ T cells expressing granzyme B exhibited closer spatial localization with tumor cells that secreted spermidine, indicating that neoplasms with elevated spermidine levels correlate with increased infiltration of GZMB^+^ CD8⁺ T cell.

### Spermidine promoted CD8⁺ T cell activation directly *in vitro*

**Figure 5:**
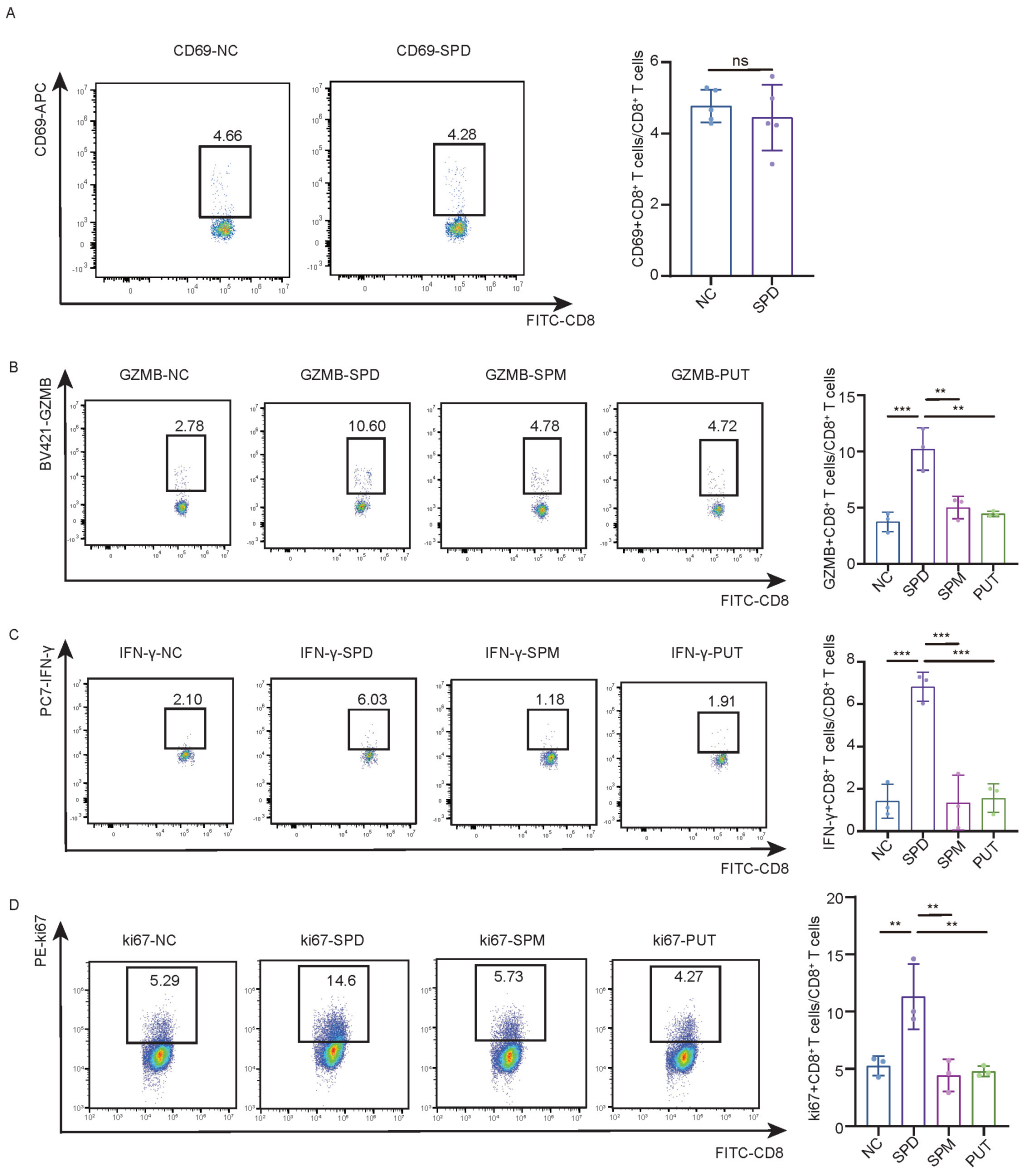
** Spermidine exerted superior efficacy in enhancing CD8⁺ T cell anti-tumor immunity. (A)**Left: representative flow cytometry results of CD69 expression in CD8⁺ T cells treated with control (NC), 10 μM spermidine (SPD), 10 μM spermine (SPM) or 10 μM putrescine (PUT) for 24 hours; right: statistical analysis of CD69 expression (n = 5). Data are expressed as mean ± SD. **(B)**Left: representative flow cytometry results of granzyme B (GZMB) expression in CD8⁺ T cells treated with control (NC), 10 μM spermidine (SPD), 10 μM spermine (SPM) or 10 μM putrescine (PUT) for 24 hours; right: statistical analysis of GZMB expression (n = 3). Data are expressed as mean ± SD. Statistical significance is denoted by “**”, indicating* p* < 0.01, “***”, indicating* p*<0.001 as determined by unpaired two-tailed Student's t-test. **(C)**Left: representative flow cytometry results of IFN-γ expression in CD8⁺ T cells treated with control (NC), 10 μM spermidine (SPD), 10 μM spermine (SPM) or 10 μM putrescine (PUT) for 24 hours; right: statistical analysis of IFN-γ expression (n = 3). Data are expressed as mean ± SD. Statistical significance is denoted by “***”, indicating* p* < 0.001 as determined by unpaired two-tailed Student's t-test. **(D)**Left: representative flow cytometry results of Ki67 expression level of CD8⁺ T cells treated with control (NC) or 10 μM spermidine (SPD) for 24 hours; right: statistical analysis of Ki67 expression (n = 3). Data are expressed as mean ± SD. Statistical significance is denoted by “****”, indicating p < 0.0001 as determined by unpaired two-tailed Student's t-test.

To further validate the effects of spermidine in activating anti-tumor immunity, naive CD8⁺ T cells were treated with spermidine for 24 hours without prior activation. Flow cytometric analysis of the surface activation marker CD69 revealed that spermidine failed to directly activate naive CD8⁺ T cells (Figure 5A). Additionally, CD3/CD28 bead-stimulated CD8⁺ T cells were treated with human polyamines, including putrescine, spermine, and spermidine, for 24 hours, followed by analysis of cytotoxic factor secretion and proliferative capacity (assessed via Ki67 expression). In CD8⁺ T cells treated with spermidine, but not spermine or putrescine, we observed significant upregulation of cytotoxic molecules, including granzyme B (GZMB) and interferon-γ (IFN-γ) (Figures 5B and 5C), and an increased Ki67⁺ cell frequency (Figure 5D). Our findings demonstratedthat spermidine maintained and enhanced CD8⁺ T cell activation following initial stimulation, concurrently strengthening cytotoxic capabilities within pre-activated CD8⁺ T cell populations.

### Spermidine supplementation potentiated anti-tumor immune responses and sensitivity to immunotherapy combined with chemotherapy *in vivo*

**Figure 6:**
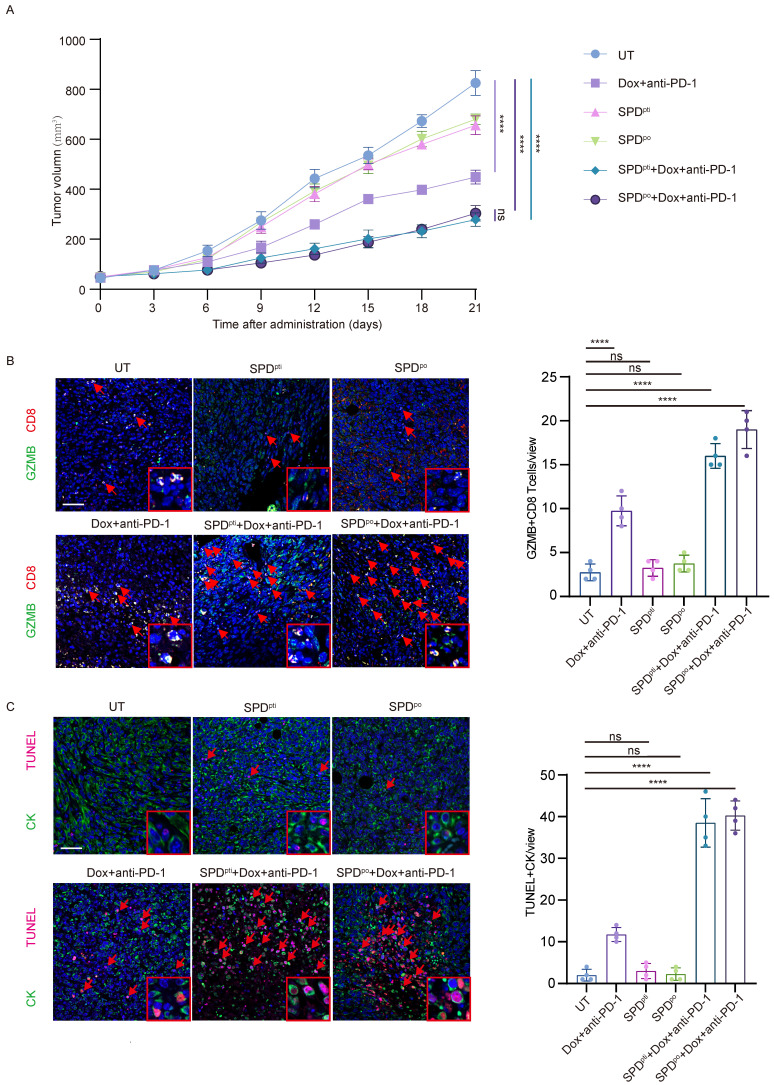
** Spermidine supplementation potentiated anti-tumor immune responses and immunotherapy sensitivity in *vivo*. (A)**Time-volume curve of tumor growth of mice in control group (UT), chemotherapy combined with immunotherapy group (Dox+anti-PD-1), peritumoral spermidine injection group (SPD^pti^), oral spermidine supplementation group (SPD^po^), chemotherapy combined with immunotherapy plus peritumoral spermidine injection group (SPD^pti^+Dox+anti-PD-1), and chemotherapy combined with immunotherapy plus oral spermidine supplementation group (SPD^po^+Dox+anti-PD-1). n = 4. **(B)**Left**:** representative images of mIHC staining for CD8 (red) and GZMB (green) in samples of mouse tumor. Scale bar: 50 μm. The detailed inset is situated in the bottom right quadrant. Right: analysis of the number of GZMB^+^ CD8^+^ T per field, n = 4. Analysis was conducted using one-way ANOVA followed by Dunnett's multiple comparison test. **(C)**Left: representative images of mIHC staining for CK (green) and TUNEL (red) in samples of mouse tumor. Scale bar: 50 μm. The detailed inset is situated in the bottom right quadrant. Right: analysis of the number of TUNEL^+^ tumor cells per field, n = 4. Analysis was conducted using one-way ANOVA followed by Dunnett's multiple comparison test.

The above data suggested that spermidine was an effective adjuvant for activating anti-tumor immunity. For further verification, we conducted xenograft mouse model using a combination of immunotherapy, chemotherapy and spermidine supplementation. After inducing EO771-luc xenografts in the fat pads of mice and randomly dividing them into a control group (no treatment), a chemotherapy combined with immunotherapy group, a peritumoral spermidine injection or spermidine oral gavage group, and a chemotherapy combined with immunotherapy plus peritumoral spermidine injection or spermidine oral gavage group. Tumor growth in mice was dynamically observed after the indicated treatment. We found that spermidine supplementation, whether administered orally or peritumorally, did not significantly impact tumor cell survival *in vivo* (Figure 6A). However, spermidine supplementation significantly enhanced the efficacy of chemotherapy combined with immunotherapy (Figure 6A). Consistent with this, immunofluorescence analysis of mouse tumor sections revealed that the spermidine-supplemented groups with chemotherapy and immunotherapy had more tumor cell death and GZMB^+^ CD8^+^ T cell infiltration (Figure 6B and 6C). These animal experiments preliminarily confirmed that spermidine as an adjuvant in immunotherapy represents a feasible therapeutic strategy, which can enhance anti-tumor immunity by promoting the activation of CD8^+^ T cells, thereby improving the efficacy of immunotherapy.

## Discussion

The TME is a complex milieu that plays a crucial role in immune responses and therapeutic efficacy 23. As an important component, tumor metabolism is involved in the formation of TME heterogeneity 24. Polyamine metabolism in solid tumors has received significant attention in recent years, as it is involved in angiogenesis 25, immunosuppression 26 and treatment resistance 26. However, as research continues, we and other researchers have found that polyamine metabolism plays a double-edged role in tumor progression 27. Previous research shows that polyamines, including spermine and spermidine, have long been associated with immunosuppression 16, 22. Spermine can directly bind to JAK1 protein, inhibiting the binding of JAK1 to related cytokine receptors, thereby blocking the activation of downstream signal transduction pathways of cytokines and inhibiting immunity 28. Meanwhile, increased spermine metabolism due to glutamine deficiency is related to impaired growth and proliferation of activation-induced T cells 29. However, supplementing aging mice with spermidine enhances the metabolic capacity of CD8^+^ T cells to produce more ATP, inhibiting immune cell senescence and thereby promoting anti-tumor immunity 30. The contradictions in the above findings regarding the effects of polyamines on immune cell function highlight the complexity of polyamine metabolism in influencing anti-tumor immunity through different pathways. This underscores the significance of in-depth research into the complex roles of spermine in tumor immunity. Our research reveals a positive correlation between robust spermidine metabolism and anti-tumor immune function mediated by tumor-infiltrating CD8^+^ T cells in breast cancer. Spermidine expressed by tumor cells can promote anti-tumor immune responses by affecting the activation of CD8^+^ T cells. This finding enriches our understanding of polyamine metabolism in regulating tumor immunity.

Immunotherapy, an emerging anti-tumor therapy in recent years, is fundamentally different from traditional therapies such as chemotherapy and radiation therapy. Its core lies in enhancing the killing ability of immune cells in the tumor microenvironment against tumor cells rather than directly targeting cancer cells 31. The advantage of this approach compared to other treatments is that once the patient's immune system is strengthened, its killing ability remains stable 32. Therefore, immunotherapy has shown promising application prospects in the treatment of breast cancer, especially triple-negative breast cancer, in recent years 33-35. However, in the clinical practice of immunotherapy for breast cancer, problems of heterogeneous treatment outcomes and drug resistance still perplex clinicians. Cancer cells may alter the immune microenvironment, causing immune cells to adopt an immunosuppressive phenotype, leading to immune escape in patients, which is deemed to be the main reason for immunotherapy resistance in breast cancer patients 36, 37. Our research confirms the important role of spermidine in regulating the activation state of CD8^+^ T cells. Importantly, we have demonstrated that supplementing spermidine can significantly enhance the efficacy of chemotherapy combined with immunotherapy *in vivo*, though it fails to directly activate anti-tumor immunity. This gives spermidine broad application prospects in tumor immunotherapy. Our research suggests that supplementing spermidine may serve as a pivotal method to promote anti-tumor immunity and enhance treatment responses in the breast cancer patients receiving chemotherapy and immunotherapy. It needs further study to reveal the mechanism of spermidine depletion in immunotherapy-resistant tumors in order to improve the reliability and stability of spermidine-supplement therapy.

In addition, a comprehensive exploration of the spermidine regulatory role in the immune microenvironment will provide a basis for the further clinical application of spermidine in the future.

Collectively, our present study demonstrates that enhanced spermidine metabolism can promote CD8^+^ T cell activation, and supplementing spermidine can significantly improve the efficacy of the combination of chemotherapy and immunotherapy. Our research provides new insights into solving the problem of immunotherapy resistance.

## Supplementary Material

Supplementary figure.
