# Supplementary figures and images for: Spermidine potentiates anti-tumor immune responses and immunotherapy sensitivity in breast cancer
Source: J Cancer. 2025 Jul 28;16(12):3684–95. doi: 10.7150/jca.113235 (PMC12435280; doi:10.7150/jca.113235)

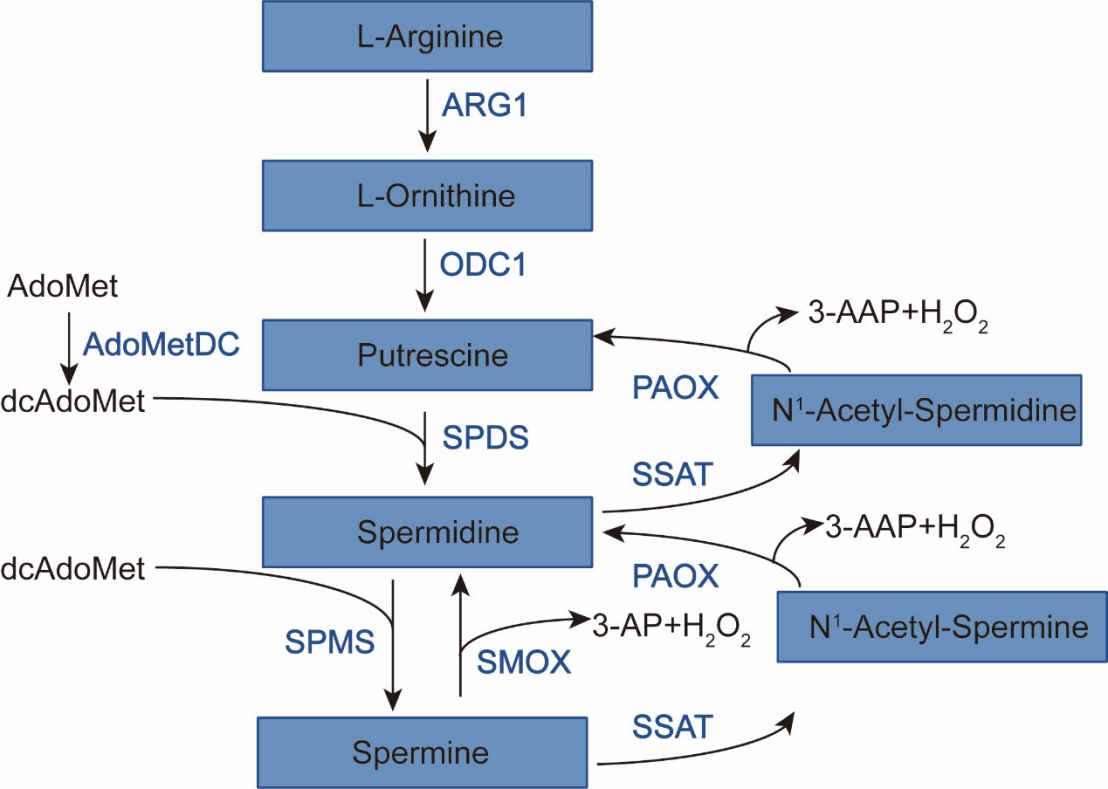

Supplement: Supplementary file 1 — Supplementary figure. [file jcav16p3684s1.pdf]
